# Characterization of Chinese Porcine Epidemic Diarrhea Virus with Novel Insertions and Deletions in Genome

**DOI:** 10.1038/srep44209

**Published:** 2017-03-09

**Authors:** Baochao Fan, Dian Jiao, Xiaona Zhao, Fengjiao Pang, Qi Xiao, Zhengyu Yu, Aihua Mao, Rongli Guo, Wanzhe Yuan, Pandeng Zhao, Kongwang He, Bin Li

**Affiliations:** 1 Institute of Veterinary Medicine, Jiangsu Academy of Agricultural Sciences, Key Laboratory of Veterinary Biological Engineering and Technology, Ministry of Agriculture, Nanjing 210014, China; 2 Jiangsu Co-innovation Center for Prevention and Control of Important Animal Infectious Diseases and Zoonoses, Yangzhou, 225009, China; 3 College of animal science and technology, Anhui Agricultural University, Hefei, 230036, China; 4 Animal, Plant and Food Inspection Center, Jiangsu Entry-Exit Inspection and Quarantine Bureau, Nanjing 210000, China; 5 College of Animal medicine, Agricultural University of Hebei, Baoding 071001, China; 6 Henan University of Animal Husbandry and Economy, Zhengzhou 450000, China

## Abstract

Outbreaks of porcine epidemic diarrhoea virus (PEDV) have caused great economic losses to the global pig industry. PEDV strains with variants in the spike (S) gene have been reported in several countries. To better understand the molecular epidemiology and genetic diversity of PEDV field isolates, in this study, we characterised the complete genome sequence of a novel PEDV variant JSCZ1601 from a outbreak in China in 2016. The PEDV isolate was 28,033 nucleotides (nt) in length without the polyadenylated sequences. Phylogenetic analysis based on the full-length genome sequence of JSCZ1601 grouped it with the pandemic variants determined post-2010 into group 2 (G2). However, the S gene of JSCZ1601 formed a new subgroup separated from the subgroups containing the other G2 strains. Comparative analysis of the amino acids encoded by the S genes revealed the N-terminal of the deduced JSCZ1601 S protein had a novel two-amino-acid deletion (N58 and S59) compared with all identified genogroups. Further, compared with the reference strains, a ‘G’ insertion was detected in the 5′ terminal of the 5′UTR of the JSCZ1601. The animal experiment revealed that this strain was high pathogenic to neonatal pigs. Taken together, a PEDV strain with the new molecular characterizations and phylogenies was found in mainland China. It is necessary to strengthen the monitoring of PEDV variations.

Porcine epidemic diarrhoea (PED) is characterised by severe watery diarrhoea, leading to dehydration and high mortality among piglets. The disease is caused by the PED virus (PEDV), which belongs to family Coronaviridae, genus Alphacoronavirus, and has an envelope surrounding a single-stranded positive-sense RNA genome^1^,^2^. The PEDV genome is approximately 28 kb nucleotides (nt) long and contains a 5′untranslated region (5′UTR), a 3′UTR with a polyadenylated tail, and at least seven open reading frames (ORFs) arranged in the following order: ORF1a, ORF1b, spike (S) glycoprotein gene, ORF3 hypothetical protein gene, envelope (E) gene, membrane (M) gene and nucleocapsid (N) gene^3^,^4^. The 5′UTR is about 291–296 nt long and the 3′UTR is about 334 nt long. Two long ORFs, ORF1a and ORF1b, occupy two-thirds of the genome and encode two nonstructural polyproteins (pp1a and pp1b) that direct genome replication and transcription^5^.

PED was first reported in feeding and fattening pigs in England in 1971^2^. In China, PED was identified as a sporadic viral enteric disease in pig herds before 2010^6^,^7^,^8^. However, outbreaks of PED with increased severity of diarrhoea, vomiting, and dehydration have occurred in China since 2010. The disease approached a mobility rate as high as 100% and a mortality rate of 80–100% in piglets less than 10 days old, and has been recognised as a devastating illness causing death in neonatal piglets^9^,^10^. New outbreaks associated with a novel PEDV strain that is genetically distant from the prototype PEDV strain, CV777, have been reported in China^8^,^11^,^12^. Since then, PED outbreaks have increased markedly and have spread rapidly across countries. In late April 2013, PED was first confirmed in the United States and subsequently spread quickly across the country^13^,^14^,^15^.

Among the proteins encoded by the ORFs, the S glycoprotein is located on the envelope of the virus where it makes up the large surface projections of the virion and plays an important role in the attachment of viral particles to the receptors of host cells^16^,^17^,^18^. Thus, the S protein is a primary target for the development of a vaccine against PED^19^,^20^. Moreover, the S protein is considered to be the most antigenic of the encoded proteins, and several investigators have reported a high degree of genetic diversity in the S glycoprotein gene^12^,^21^,^22^. According to the phylogenetic analyses of the spike S gene, the PEDV strains were divided into two distinct clusters designated genogroup 1 (G1; classical) and genogroup 2 (G2; field epidemic or pandemic)^21^,^23^. Concurrently, several variant PEDV strains, characterised by insertions and deletions (INDELs) in the S gene and designated S-INDEL PEDV, were found to be circulating in United States swine farms^24^,^25^. Several strains with different deletions in the S gene were also reported in previous studies^22^,^26^,^27^,^28^,^29^,^30^. Thus, the S gene may be important for understanding the genetic relatedness of PEDV field isolates, the epidemiological status of the virus, and for vaccine development.

Thereby, a comprehensive study is necessary to better understand the genetic variations and relationships between different strains, and would be helpful to find out the reason of the continuously outbreak of PEDV and develop new strategy to control and prevent PEDV infection. In this study, we found a novel PEDV field strain JSCZ1601 with amino acid deletions in the protein encoded by the S gene, and a ‘G’ insertion in the 5′ terminal of the 5′UTR. The animal experiment revealed that this strain was high pathogenic to neonatal pigs. This study showed that a PEDV strain with the new molecular characterizations and phylogenies was found in mainland China. This will be useful to take into consideration in the control and prevention of this disease.

## Results

### Full-length genome sequence analysis

The sequence analysis showed that the full-length genomic sequence of JSCZ1601 (accession no. KY070587) was 28,033 nt long, excluding the polyadenylated sequences, and included the following genes and UTRs: 5′UTR (293 nt), ORF1a (12,354 nt), ORF1b (8,037 nt), S (4,155 nt), ORF3 (675 nt), E (231 nt), M (681 nt), N (1,326 nt), and 3′UTR (334 nt). We compared the full-length JSCZ1601 genome with the genomes of the reference PEDV isolates shown in Table 1. The comparative analyses showed that the nucleotide identities between JSCZ1601 and the strains identified post-2010 (e.g., Chinese strains JS-HZ2012, GD-A, and AH2012, and American strains PC21A, USA/Minnesota61/2013, and USA/Michigan252/2014) ranged from 98.0–99.0%, while the nucleotide identities with genomes determined ante-2010 (CV777, JS2008, DR13, and attenuated DR13) ranged from 96.6–97.4%.

The full-length genome sequence of JSCZ1601 shared the highest levels of nucleotide identity (99.0%) with PEDV strains PEDV-LYG, JS-HZ2012, and YC2014. However, among the ORFs, the S genes had the lowest nucleotide identities 98.2–98.4% with these strains. The amino acid identity between the deduced S proteins of JSCZ1601 and PEDV-LYG was only 97.6%. These data indicate that a high level of variation may have occurred in the S protein of the JSCZ1601 strain. The deduced ORF1b protein of JSCZ1601 shared ≥99.3% amino acid identities with all the reference strains except AH2012, which shared only 94.3% identity. The 5′UTR of JSCZ1601 shared 83.2–98.9% identities with the other PEDV strains. The 3′UTR of JSCZ1601 showed higher genetic conservation than the 5′UTR and shared 94.5–99.1% homology with the other PEDV stains. Besides, the identities shared between the 5′UTR of JSCZ1601 and the three American strains (USA/Minnesota61/2013, USA/Michigan252/2014 and USA/Iowa106/2013) were only 83.2–86.6%.

### Phylogenetic analyses

**Figure 1:**
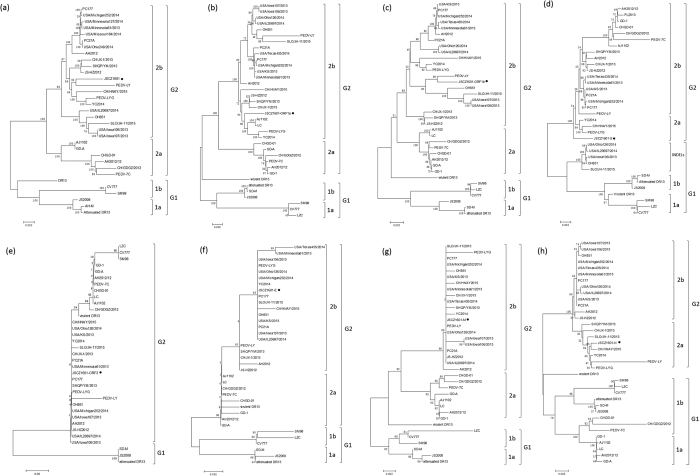
Phylogenetic analyses of PEDV strains based on the nucleotide sequences of the full-length genome and the ORF1a, ORF1b, S, ORF3, E, M, and N genes. Phylogenetic trees of (**a**) the full-length genome; (**b**) the ORF1a gene; (**c**) the ORF1b gene; (**d**) the S gene; (**e**) the ORF3 gene; (**f**) the E gene; (**g**) the M gene; (**h**) the N gene. The evolutionary history was inferred using the neighbour-joining method. The percentage of replicate trees in which the associated taxa clustered together in the bootstrap test (1000 replicates) are shown next to the branches. The evolutionary distances were computed using the Jukes-Cantor method and are presented as the number of base substitutions per site. The evolutionary analyses were conducted using MEGA5 software.

Phylogenetic trees were generated based on the nucleotide sequences of the full-length genomes and each of the seven ORFs (i.e., ORF1a, ORF1b, S, ORF3, E, M, and N) of JSCZ1601 and the other PEDV strains (Fig. 1a–h, respectively). As shown in Fig. 1a, the phylogenetic tree of the full-length genome indicated that the PEDV strains fell into two groups designated G1 and G2. G1 had two subgroups, 1a and 1b, and consisted of CV777, virulent and attenuated DR13, SM98, and an earlier Chinese strain JS2008. G2 also had two subgroups, 2a and 2b (Fig. 1a). Notably, all the strains identified from 2011 to 2015 were in G2. The phylogenetic trees of the full-length genome, ORF1a, ORF1b, E, and M showed that JSCZ1601 fell into G2b. However, the S gene of JSCZ1601 formed a sole subgroup that was separate from the other G2 strains (Fig. 1d). These data indicate that significant variation had occurred in the S gene of JSCZ1601. Moreover, the phylogenetic tree of the S genes revealed that the S-INDEL strains formed a single subgroup in G1.

### Comparative analysis of the deduced amino acid sequences of complete S genes

**Figure 2:**
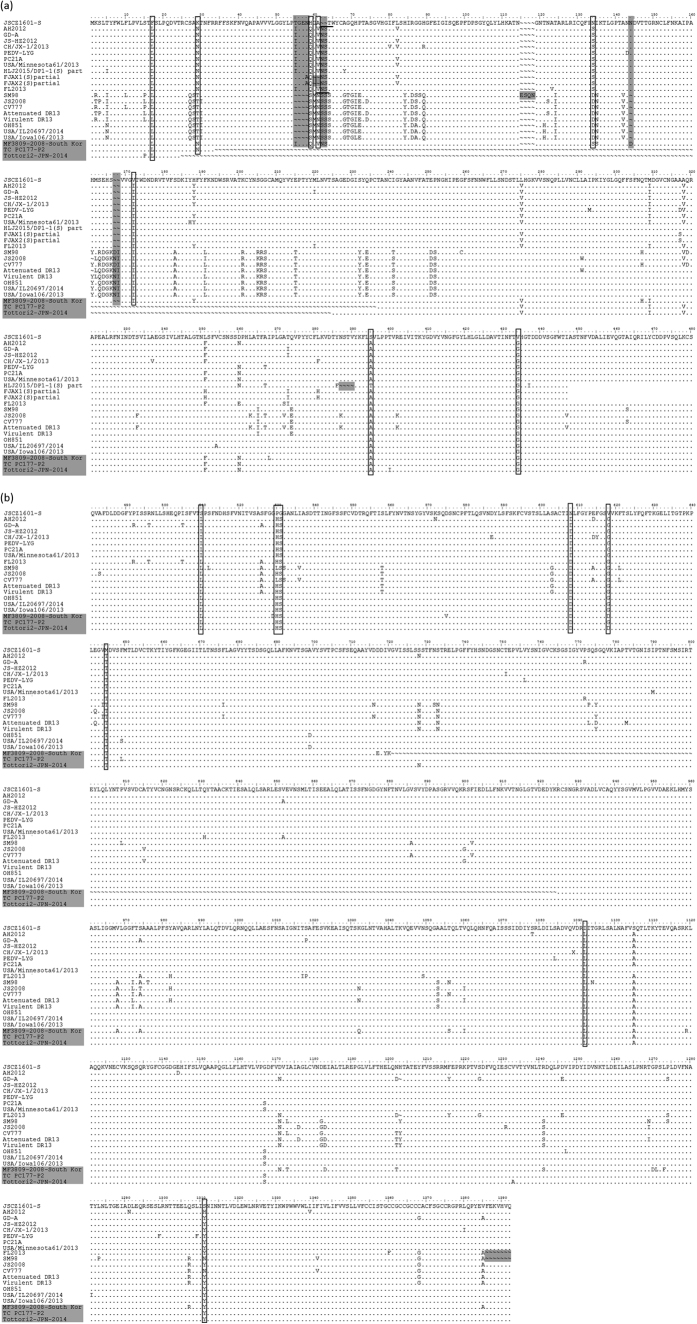
Alignment of the deduced amino acid sequences of the complete S proteins of JSCZ1601 and several representative PEDV strains. Deletions and insertions are shown as grey bars. Key amino acid mutations within the different strains are shown as black boxes, and the N-glycosylation sites are indicated by short lines. (**a**) Alignment of the sequences of all the selected PEDV strains. (**b**) Alignment after removing three PEDV strains, FJAX1, FJAX2, and HLJ2015/DP1-1, in which the S genes were partial.

As shown in Fig. 2, compared with the S protein sequence of G1 strain CV777, the deduced S protein of JSCZ1601 had two insertions (55TGENV58 and 136 N) and one deletion (157NI158), which is the same as the S proteins of the G2 strains AH2012, GD-A, and PEDV-LYG. Notably, the N-terminal of the JSCZ1601 S protein had an additional two-amino-acid deletion (N58 and S59) compared with the S protein of the representative strain CV777. And this deletion was also existed in JSCZ1601 compared with G2 strains, such as AH2012 and GD-A. Further, the two-amino-acid deletion in the JSCZ1601 S protein eliminated a potential N-glycosylation site that was located at positions 62–64 and 57–59 in the amino acid sequences of the S proteins in AH2012 and CV777, respectively. An additional 16 unique amino acid substitutions were detected in the S protein of JSCZ1601 compared with the S proteins of all the reference strains (Fig. 2). In addition, five unique substitutions (500 L/S, 520 H/P, 521 S/G, 590 D/N, and 600 G/V) occurred in one neutralizing epitope (amino acids 499–638) of the JSCZ1601 receptor-binding subunit S1 protein compared with all the reference strains. Moreover, the deduced S proteins of three S-INDEL strains, OH851, USA/IL20697/2014, and US/Iowa106/2013, had the same amino acid deletions and insertion as the G1 strains CV777 and JS2008.

### Nucleotide sequence comparison of 5′-RNA termini

**Figure 3:**
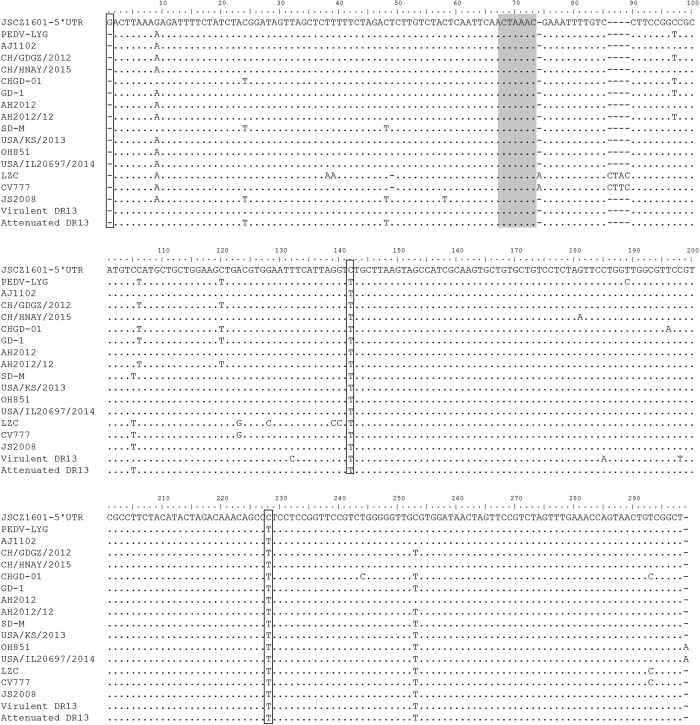
Alignment of nucleotide sequences of the 5′UTRs of JSCZ1601 and several representative PEDV strains. Deletions and key nucleotide mutations within the different strains are shown as black boxes. The core sequences (5′-CUAAAC-3′) of the leader transcription-regulating sequences (CS-L) are shown as grey bars.

The 5′UTR of JSCZ1601 was 293 nt long, which is 3-nt shorter than the 5′UTR of CV777. As shown in Fig. 3, 1-nt deletion, 4-nt deletions, and 1-nt insertion were detected in the proximal regions of the 5′UTRs of the CV777 and LZC strains compared with the other strains. Conversely, the 5′UTRs were relatively conserved between G2 members. Interestingly, a ‘G’ insertion was observed in the 5′ terminal of the 5′UTR of JSCZ1601. And two another nucleotide substitutions, 137 C/T and 225 C/T were existed in the 5′UTR of JSCZ1601, when compared with the reference strains. Besides, the core sequence (5′-CUAAAC-3′) of the PEDV leader transcription-regulating sequence was well conserved with no nucleotide substitutions detected in any of the PEDV strains.

### The pathogenesis of JSCZ1601 in newborn piglets

To determine the pathogenicity of JSCZ1601 in newborn piglets, suckling piglets were orally inoculated with the inocula prepared by the intestine tissue sample. While control animals were inoculated with PBS.

**Figure 4:**
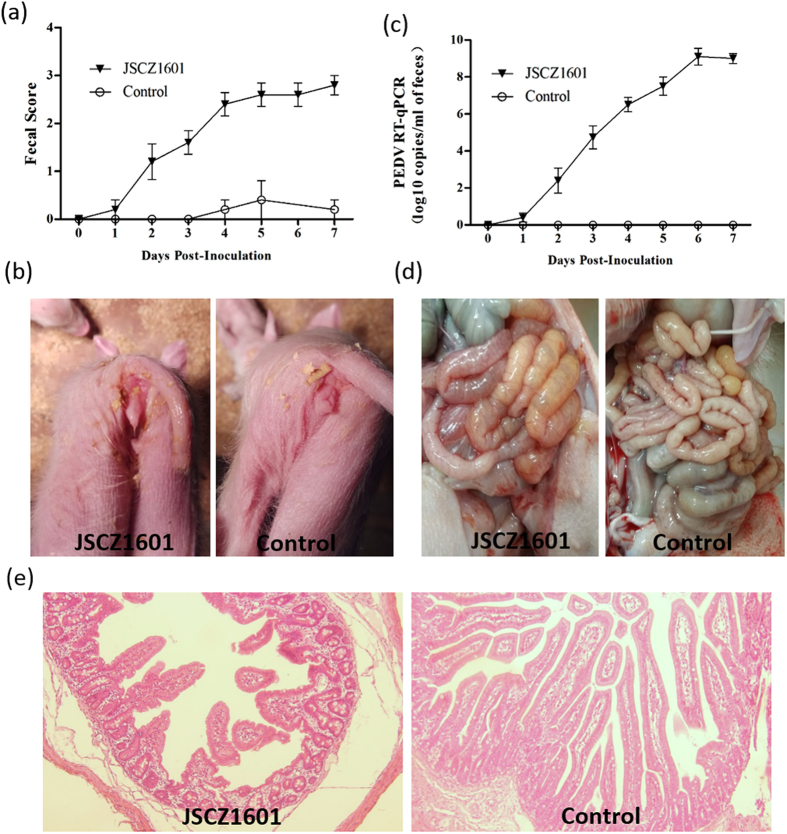
The pathogenesis of JSCZ1601 in newborn piglets. (**a**) Mean fecal scores after viral inoculation. (**b**) The diarrhea symptoms of the piglet inoculated with JSCZ1601. (**c**) Mean RT-qPCR titers of the fecal samples. (**d**) Macroscopic examinations of the intestine of piglets inoculated with JSCZ1601 and control medium. (**e**) HE-stained small intestines of piglets inoculated with JSCZ1601 and control medium.

During the acclimation period, the clinical signs were monitored and piglets in the JSCZ1601 challenge group exhibited lethargy and diarrheic feces by 2 day post-inoculation (DPI) and diarrhea lasted through the study period (Fig. 4a and b). The piglets in the negative control group remained active and clinically unaffected throughout the 7-day study period. Due to severity of clinical signs, these piglets were euthanized and necropsied at 7 DPI. The quantitative genomic copies/ml of PEDV RNA in fecal samples rectal was shown in Fig. 4c. The average virus shedding in JSCZ1601 group increased about 10^2.5^ to 10^9.5^ genomic copies/ml through the study period. Negative control piglets remained active with normal feces and fecal shedding of PEDV remained undetected throughout the study period.

Necropsy examinations showed that the virus inoculated piglets displayed typical PED-like lesions. The small intestine was thin-walled and contained soft to watery contents (Fig. 4d). Small intestine contents obtained at necropsy were also tested by PEDV RT-qPCR and had 10^7.2^ to 10^10.1^ genomic copies/ml. Histopathological examination showed severe necrosis and villous atrophy of the small intestinal enterocytes in JSCZ1601 inoculated pigs (Fig. 4e). Villus height and crypt depth were measured, and the mean villous height/crypt depth (VH/CD) ratio of the mock-inoculated piglets (6.5±0.8) was higher than those of JSCZ1601inoculated piglets (1.3±0.5).

## Discussion

In the winter of 2010, a PED outbreak began on pig farms in southern China and immediately spread throughout the country. The death toll from the disease was over one million piglets in South China, with devastating damage to the pig industry. Our genetic analyses showed that the PEDV strains fell into two genogroups, G1 (classical) and G2 (variant)^21^,^23^. The prevalent strains that caused the outbreak in Asia in late 2010 and the recent outbreaks in North America belonged to the G2 genogroup^13^,^31^. In this study, we found a PEDV strain JSC1601 with new genetic characteristics while elucidating the molecular characteristics and phylogeny of PEDV field strains in China. The whole-genome phylogenetic analysis demonstrated that JSCZ1601 along with the strains determined post-2010 clustered into genogroup G2. However, the S gene of JSCZ1601 formed a new subgroup that was separated from the other G2 strains. Further, comparative analysis of the amino acid sequence of the S protein revealed that the N-terminal of the JSCZ1601 S protein had a two-amino-acid deletion (N58 and S59) compared with the other G2 members. Compared with the other classical and variant strains, a ‘G’ insertion was also observed in the 5′ terminal of the JSCZ1601 5′UTR. These data revealed that a PEDV strain with the new molecular characterizations and phylogenies was found in mainland China.

Previous studies have demonstrated that the 5′UTR of PEDV strains were highly conserved^7^,^13^,^32^. In general, strict conservation of these regions is essential for the PEDV life cycle. It is known that the 5′UTRs of coronaviruses form conserved RNA structural elements that are critical for viral replication, subgenomic mRNA transcription, and translation^33^. In this study, we found a ‘G’ insertion in the 5′ terminal of the 5′UTR compared with the other PEDV strains. Studies on the beta-coronavirus indicated that stem-loop 4 (SL4) in the 5′UTRs was conserved among group 2 coronaviruses and may have a homolog in groups 1 and 3, and that SL4 may act as a cis-acting element for defective interfering RNA replication through interactions with cellular proteins^34^. In this study, the 223 T/C mutation was located in the SL4, which was identified in the 168–297nt region^34^. How these insertion and mutations affect the efficiency of virus replication needs to be addressed in future studies. The core sequence (5′-CUAAAC-3′) in the transcription-regulating sequence of PEDV, which was also present in JSCZ1601, was reported to be a determinant factor in transcriptional regulation in coronavirus because the synthesis of subgenomic mRNA requires the appropriate tertiary structure of the core sequence^35^.

The S protein of PEDV is known to play pivotal roles in viral entry and in inducing the neutralizing antibodies in natural hosts, and this makes it a primary target for the development of effective vaccines against PEDV^17^,^22^,^36^,^37^. Significant genetic variations in the S gene have been revealed between the newly determined PEDV field strains and early isolates^21^,^38^. In this study, the amino acid sequence of the JSCZ1601 S protein shared only 97.6–98.1% identities with the S proteins of the reference strains. Besides, the phylogenetic analyses showed that the S gene of JSCZ1601 formed a separated subgroup in G2. All these data implied that the S gene of JSCZ1601 had undergoing significant genetic changes. The spike ectodomain consists of a receptor-binding subunit S1 and a membrane-fusion subunit S2. S1 contains two domains, an N-terminal domain and a C-terminal domain, both of which can potentially function as receptor-binding domains^39^,^40^. Several neutralizing epitopes (499–638, 748–755, 764–771, and 1,368–1,374 amino acids) have also been identified^17^,^36^,^41^. In this study, the deduced JSCZ1601 S protein had a two-amino-acid deletion (N58 and S59) in the S1 N-terminal domain. And this deletion completely eliminated a potential N-glycosylation site that was present in the S proteins of the other strains. Moreover, five unique amino acid substitutions were identified in one neutralizing epitope of the S1 C-terminal domain of the JSCZ1601 S protein compared with the S proteins of all the PEDV reference strains. Whether these amino acids substitutions and the N-glycosylation site deletion influence the antigenicity and pathogenicity of PEDV remains to be investigated.

**Figure 5:**
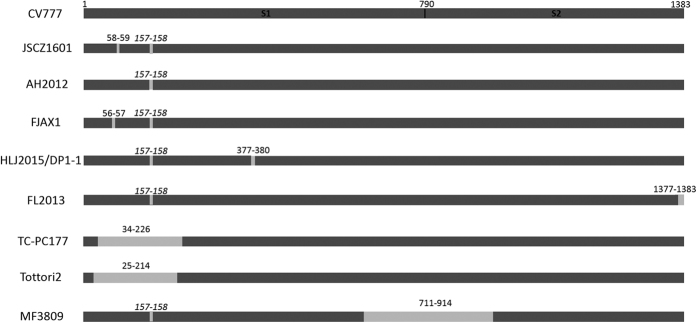
Simple schematic diagram of the deduced S proteins of PEDV strains with the new deletions. The reference strain was CV777. Deletions are shown as grey bars. The numbers indicate the positions of the deletion according to the CV777 sequence. Deletions that have been identified in G2 strains are labelled with italicised numbers.

In this study, we also summarized the deletions in the S genes in the reported strains and illustrated the results in a simple schematic diagram (Fig. 5). Three strains, which has a large deletion in S gene, MF3809 with 711–914 amino acid deletion^42^, TC PC177 with 34–226 amino acid deletion^27^ and Tottori2-JPN-2014 with 25–214 amino acid deletion^26^ compared with the reference strain CV777 (Fig. 5). This finding suggested that positions 25–226 in subunit S1 and 711–914 in subunit S2 of the spike ectodomain may be non-essential regions in PEDV infection. The pathogenicity of the Tottori2-JPN-2014 strain has been reported to be mild^26^. Further, the novel PEDV strains with new amino acid deletions in their S genes have been found in fields in China. The S proteins of strains FJAX1 and FJAX2 were found to have a 66–67 amino acid deletion^28^ and the S protein of HLJ2015/DP1–1 had a 377–380 amino acid deletion^29^ compared with the CV777 S protein. However, the pathogenicity of these three strains has not been reported so far. A Chinese variant strain FL2013, which had a seven amino acid deletion (FEKVHVQ) in the C-terminus of the deduced S protein compared with the S proteins of the other G2 PEDV sequences, had reduced virulence in newborn piglets^30^. Together, these findings revealed that the genetic evolution of PEDV strains was variant, and the S gene of PEDV might be an important virulence gene. It is important to monitor the genetic variations in this virus, and virulent genes and regions of the PEDV genomes should be identified using the reverse genetic technologies. In addition, we also have investigated 35 samples from different pig farms in different cities and provinces (data not shown), and only one pig farm was found to have the epidemic of this strain JSCZ1601. These data indicated that the strain had a certain ability to spread, and the positive monitoring and prevention of epidemic of the strain is necessary.

The intestinal tissue samples of strain JSCZ1601 were obtained from suckling piglets less than 10 days old with severe watery diarrhoea, vomiting, and dehydration in a large-scale pig farm in Changzhou, Jiangsu Province, China. A severe diarrhoea epidemic broke out on this farm in January 2016 and caused the death of hundreds suckling piglets less than one week old. Several important pathogens, such as TGEV, RV, PRRSV, PCV, CSFV, and PRV, were confirmed to be negative in these samples (data not shown). We also checked several intestinal samples obtained from different piglets from different sows. The S gene sequences of these samples had the same characteristics as the JSCZ1601 S gene, which confirmed the severe diarrhoea epidemic was caused by the PEDV strain JSCZ1601. In order to measure the pathogenesis of JSCZ1601 in newborn piglets further, we used the virus inocula prepared from the intestine tissue sample and found that the JSCZ1601 strain could cause severe watery diarrhoea in piglets. And the low challenge dose (3 ml 1.42×10^4^copies/ml inocula each piglet) might be one reason for the delayed time of diarrhea and the longer duration of disease. These results also revealed that the two-amino-acid deletion in the S protein did not attenuate the pathogenicity of the JSCZ1601 strain. We tried to isolate this strain *in vitro* using different Vero cell lines with trypsin, but failed. We will make attempts to obtain the virus isolate further.

In conclusion, here we reported the genetic characterisation of a novel PEDV strain, JSCZ1601, found on the Chinese mainland, which had significant variations in the S gene. In recent years, new S mutant strains have been constantly found and this may be related to the immune pressure of the vaccines that are used, including those from vaccine strains CV777 and DR13. Meanwhile, the virulence of the new mutants may be changed, and amino acid substitutions in the neutralizing epitopes may have conferred the capacity for immune evasion in these PEDV field strains. The monitoring of PEDV variations will be useful in the control and prevention of PED.

## Methods

### Clinical samples

The intestinal tissue sample was obtained from a < 10-day-old suckling piglets with severe watery diarrhea, vomiting and dehydration in a large-scale pig farm with severe diarrhea epidemic in Changzhou, Jiangsu province, China in 2016. The piglet was diagnosed as PEDV-positive using reverse transcription (RT) and polymerase chain reaction (PCR). In addition, the possible co-infections caused by porcine transmissible gastroenteritis virus (TGEV), porcine rotavirus (PRV), and other common pathogenic intestinal pathogens, had been confirmed to be negative (data not shown).

### Complete Genome sequencing

Total RNA was extracted from the tissue homogenate using the RNeasy Mini Kit (Qiagen, Hilden, Germany) according to the manufacturer’s instructions, and suspended in nuclease-free water immediately before use. The first-strand cDNA synthesis was performed using the SuperScript III First-Strand Synthesis Kit (Invitrogen, Carlsbad, CA, USA) following the manufacturer’s protocols.

The entire PEDV genome was amplified by 14 pairs of primer designed with Primer 5.0 software based on the conserved regions determined by a multiple alignment analysis of the reference strains and CV777, and the 5′ and 3′ termini of the genomic sequence were synthesized by using the rapid amplification of the cDNA ends (RACE) kit (Clontech, Beijing, China) following the manufacturer’s instructions. The sequences of the primers used for the whole genome and the RACE procedure are provided in Table 2. The overlapping fragments were amplified by PCR using the Phanta Super Fidelity DNA polymerase (Vazyme, China), and the thermal cycling was performed at 95 °C for 3 min, followed by 35 cycles of 95 °C for 15 s, 52 °C to 56 °C for 15 s, and 72 °C for 2 min, with a final extension at 72 °C for 3 min. The amplified PCR products were analyzed using agarose gel electrophoresis. The PCR products were purified from the agarose gel using the AxyPrep DNA Gel Extraction Kit (Axygen, China), and cloned into the pEASY-Blunt Zero vector (Trans, China). Three clones were sequenced by a commercial service provider (Invitrogen, Shanghai, China).

### Nucleotide and amino acid sequence analyses

The overlapping sequences of the PCR products were combined to obtain the full-length genomic sequence of the JSCZ1601 strain. A nucleotide BLASTn analysis was used to compared the sequences of the JSCZ1601 genes with those of the reference strains of PEDV in the GenBank database (Table 3). The sequence alignments were generated using the Clustal W program. Phylogenetic trees of the full-length genomic, ORF1a, ORF1b, S, ORF3, E, M and N nucleotide sequences were generated using the distance-based neighbor-joining method in the MEGA, version 5.05, software. The bootstrap values were calculated based on 1000 replicates, and the evolutionary distances were computed using the Jukes-Cantor method. The deduced amino acid sequence comparisons were obtained using the BioEdit software.

### Experimental design of infection

For the preparation of inocula for piglets, the intestine tissue sample was homogenized, diluted 1:4 in phosphate-buffered saline (PBS, pH 7.4; Sigma-Aldrich, St. Louis, MO), and centrifuged. The supernatant was filtered through a 0.45-μm-pore-size syringe filter (Millipore, Billerica, MA, USA) and collected as the inocula. The RNA titers of the inocula was 1.42 log10 genomic copies/mL by using quantitative real-time reverse transcription-PCR (RT-qPCR) as reported^43^.

Eight 3-day-old piglets, selected from one sow that was PEDV RNA and antibody negative, were divided into two groups (four piglets each) and housed in separate rooms. Pigs were fed a mixture of liquid milk replacer and yogurt and had free access to water. Groups A was challenged orally with 3 ml inocula. The four control piglets (Group B) were inoculated with PBS. All animals were monitored daily for clinical signs of disease, including diarrhea and vomiting. Rectal swabs were collected for scoring fecal denseness (scores: 0 normal; 1 pasty stool; 2 semiliquid diarrhea; and 3 liquid diarrhea) and for enumerating fecal viral RNA shedding by RT-qPCR. At necropsy, intestinal tissues and contents were grossly evaluated. Additionally, a portion of the jejunum and ileum were fixed in 10% neutral buffered formalin for histopathology. The villous atrophy is a typical histopathological change of the infection of a highly virulent PEDV^44^,^45^,^46^,^47^. And the villous height and crypt depth (VH:CD) ratios would be calculated using a computerized image system with as previous described^44^. All of the animal experiments were performed with the approval of the Jiangsu Academy of Agricultural Sciences Experimental Animal Ethics Committee (NKYVET 2015–0127) and were performed in accordance with relevant guidelines and regulations. All efforts were made to minimize animal suffering and to reduce the number of animals used.

### Statistical analysis

All data were analyzed using GraphPad Prism (Version 5.03, San Diego, CA, USA) software. Differences among groups were examined using one-way analysis of variance (ANOVA), followed by Tukey’s tests.

## Additional Information

**How to cite this article:** Fan, B. *et al*. Characterization of Chinese Porcine Epidemic Diarrhea Virus with Novel Insertions and Deletions in Genome. *Sci. Rep.* **7**, 44209; doi: 10.1038/srep44209 (2017).

**Publisher's note:** Springer Nature remains neutral with regard to jurisdictional claims in published maps and institutional affiliations.

## Figures and Tables

**Table 1:** Nucleotide and amino acid sequence identities (%) of different regions of the JSCZ1601 genome compared with those of the other viruses.

	Identity to JSCZ1601/ Nucleotide (Amino Acid)									
strains	Complete genome	5′UTR	ORF1a	ORF1b	S	ORF3	E	M	N	3′UTR
PEDV-LYG	99.0 (−)	97.2 (−)	99.1 (99.1)	98.5 (99.3)	**98.3** (**97.6)**	100 (100)	100 (100)	99.5 (100)	99.4 (99.3)	95.9 (−)
JS-HZ2012	99.0 (−)	97.9 (−)	99.0 (99.1)	98.9 (99.7)	**98.2** (**98.0)**	99.4 (100)	99.5 (100)	100 (100)	98.7 (99.0)	95.0 (−)
YC2014	99.0 (−)	91.0 (−)	99.2 (99.2)	98.6 (99.4)	**98.4** (**98.1)**	100 (100)	100 (100)	99.8 (100)	99.5 (99.5)	95.4 (−)
GD-A	98.5 (−)	96.9 (−)	97.8 (98.3)	98.6 (99.6)	96.8 (96.8)	95.8 (96.4)	99.1 (100)	98.5 (97.7)	95.8 (96.8)	95.6 (−)
AH2012	98.8 (−)	98.6 (−)	99.0 (99.2)	**93.8** (**94.3)**	97.9 (97.5)	98.6 (99.5)	99.1 (100)	99.7 (100)	98.4 (98.6)	95.0 (−)
AH2012/12	98.0 (−)	97.2 (−)	98.1 (98.5)	98.6 (99.6)	96.7 (96.5)	95.7 (96.4)	99.1 (100)	98.5 (98.2)	95.9 (96.8)	96.5 (−)
CHGD-01	98.0 (−)	96.2 (−)	98.0 (98.3)	98.5 (99.4)	96.9 (96.8)	96.2 (97.3)	98.7 (98.7)	98.3 (98.2)	95.9 (96.8)	98.5 (−)
CH/JX-1/2013	98.9 (−)	98.9 (−)	98.9 (98.9)	98.8 (99.6)	97.9 (97.4)	99.7 (100)	99.5 (100)	99.8 (100)	99.0 (99.0)	99.1 (−)
JS2008	96.8 (−)	96.9 (−)	97.3 (97.7)	98.0 (99.4)	93.5 (92.5)	38.0 (37.7)	96.1 (94.8)	97.7 (97.3)	95.7 (96.8)	95.3 (−)
CV777	96.6 (−)	95.3 (−)	96.7 (97.3)	97.7 (99.3)	93.5 (92.8)	96.8 (96.4)	96.9 (98.7)	98.2 (98.6)	95.3 (96.3)	95.0 (−)
DR13	97.4 (−)	97.6 (−)	97.6 (98.1)	98.3 (99.7)	94.4 (93.8)	98.5 (99.5)	98.2 (100)	98.3 (98.6)	96.5 (96.8)	94.5 (−)
Attenuated_DR13	96.7 (−)	97.6 (−)	97.3 (97.7)	97.8 (99.5)	93.3 (92.4)	38.0 (37.7)	87.8 (87.0)	97.9 (97.7)	95.7 (96.8)	95.0 (−)
PC21A	98.7 (−)	91.1 (−)	98.7 (99.1)	98.5 (99.5)	98.4 (97.9)	100 (100)	100 (100)	100 (100)	98.8 (99.0)	93.3 (−)
USA/Minnesota61/2013	98.7 (−)	**86.6** (−)	99.1 (99.4)	98.7 (99.7)	98.4 (97.9)	100 (100)	99.5 (100)	100 (100)	98.8 (99.0)	94.5 (−)
USA/Michigan252/2014	98.7 (−)	**83.2** (−)	98.8 (99.1)	98.6 (99.5)	98.3 (97.7)	100 (100)	100 (100)	100 (100)	98.8 (99.0)	95.9 (−)
USA/Iowa106/2013	98.3 (−)	**86.6** (−)	98.8 (99.1)	98.9 (99.8)	96.1 (95.3)	100 (100)	99.5 (100)	99.7 (100)	98.7 (98.8)	94.5 (−)

**Table 2:** The primers of 14 overlapping fragments used for amplification of JSCZ1601 whole genome.

Primers name	Sequence
186-2055-F	5′-GCGTTCCGTCGCCTTCTACATAC-3'
186-2055-R	5′-TTATAAACAGGATGTTCAATGA-3'
1960-4005-F	5′-CAGTTGTTGTTGATGGACTTGC-3'
1960-4005-R	5′-CCACTATCATTGCCTATAAAAG-3'
3925-5998-F	5′-TCGAGATACTACTGCTCTCTCC-3'
3925-5998-R	5′-TCCATCATACACCATACCAGTG-3'
5922-7969-F	5′-CATTCCTAGATAATGGTAACGG-3'
5922-7969-R	5′-ATCATAATCGCTATCACTGCTA-3'
7893-9941-F	5′-TGTTCATAGTTGCTGTTTTCTT-3'
7893-9941-R	5′-TAAGCCACCAAGTAGAACCATT-3'
9869-11925-F	5′-AGTAGTCTGTTTACGGAGAATG-3'
9869-11925-R	5′-ATGCCATCTCCTTCTGCCTTAA-3'
11845-13885-F	5′-GCGTATTGTCAAGCTCCAGAAT-3'
11845-13885-R	5′-AAGTAAGCTCAGAGCCCTCAGA-3'
13808-15857-F	5′-AACCTGGCCATTTCAATAAGGA-3'
13808-15857-R	5′-TCTTTAGGTCCTACAACCTCAT-3'
15781-17829-F	5′-ACTATCAAGGCCAAGGAGGAGA-3'
15781-17829-R	5′-CGTATGCAGCGCACTATTGTAA-3'
17759-19825-F	5′-TCAAGATTGGACCAAGTAAGAG-3'
17759-19825-R	5′-CAGCACCATAGTTATAGAGATT-3'
19750-21810-F	5′-GGTTATTCCATGCCTTCTATTT-3'
19750-21810-R	5′-GAGGTAAAACAGCCAAGAATTT-3'
21729-23770-F	5′-GCTATCCAAGTACCCTATTATTG-3'
21729-23770-R	5′-CCCTGCGAATTAACAACCTCTT-3'
23707-25755-F	5′-CTAAGGGTTTGAACACTGTGGC-3'
23707-25755-R	5′-GATATTCCATGTGAAATTCCAG-3'
25688-27848-F	5′-ATGTCTAACGGTTCTATTCCCG-3'
25688-27848-R	5′-CCACTGGCTTACCGTTGTGTGC-3'
5′ RACE Primer (303-284)	5′-TTGCTAGCCATAGCCGACAG-3'
3′ RACE Primer (27724-27746)	5′-CTATGTCCCAGGGTAGTGCCATT-3'

The location corresponds to position within the JS-HZ2012 (KC210147) genome.

**Table 3:** Representative PEDV strains used in this study.

Strain	Country	Year	Accession No.	Strain	Country	Year	Accession No.
PEDV-LYG	China	2014	KM609212	CV777	Europe		JN599150
JS-HZ2012	China	2012	KC210147	Virulent DR13	South Korea	2009	DQ862099
YC2014	China	2014	KU252649	Attenuated_DR13	South Korea		JQ023162
GD-A	China	2012	JX112709	SM98	South Korea	2010	GU937797
LZC	China	2006	EF185992	MF3809	South Korea	2008	KF779469
JS2008	China	2008	KC109141	Tottori2-JPN-2014	Japan	2014	LC022792
CHGD-01	China	2011	JX261936	SLO/JH-11/2015	Slovenia	2015	KU297956
PEDV-7C	China	2011	KM609204	PC21A	USA	2013	KR078299
GD-1	China	2011	JX647847	USA/Minnesota61/2013	USA	2013	KJ645705
AJ1102	China	2011	JX188454	USA/KS/2013	USA	2013	KJ184549
LC	China	2011	JX489155	USA/Minnesota52/2013	USA	2013	KJ645704
CH/CG/11	China	2011	JQ627654	PC177	USA	2013	KR078300
CH8	China	2011	JQ239436	USA/Iowa106/2013	USA	2013	KJ645695
AH2012	China	2012	KC210145	USA/Iowa107/2013	USA	2013	KJ645696
AH2012/12	China	2012	KU646831	TC PC177	USA	2013	KM392229
CH/GDGZ/2012	China	2012	KF384500	USA/IL20697/2014	USA	2014	KT860508
AH2012/12	China	2012	KU646831	USA/Michigan252/2014	USA	2014	KR265822
CH/AHHF-2/2012	China	2012	JX018182	USA/Texas435/2014	USA	2014	KR265834
FL2013	China	2013	KP765609	USA/Ohio126/2014	USA	2014	KJ645702
CH/JX-1/2013	China	2013	KF760557	OH851	USA	2014	KJ399978
SHQP/YM/2013	China	2013	KJ196348	USA/Iowa303/2014	USA	2014	KR265827
CH/HNAY/2015	China	2015	KR809885	JSCZ1601	China	2016	KY070587
